# Force spectroscopy-based simultaneous topographical and mechanical characterization to study polymer-to-polymer interactions in coated alginate microspheres

**DOI:** 10.1038/s41598-019-56547-z

**Published:** 2019-12-27

**Authors:** Maria Virumbrales-Muñoz, Edorta Santos-Vizcaino, Laura Paz, Amparo Maria Gallardo-Moreno, Gorka Orive, Rosa Maria Hernandez, Manuel Doblaré, Maria Luisa Gonzalez-Martin, Luis Jose Fernández, Jose Luis Pedraz, Ignacio Ochoa

**Affiliations:** 1 Department of Biomedical Engineering, University of Wisconsin, Madison, WI USA; 2 NanoBioCel Group, Laboratory of Pharmaceutics, School of Pharmacy, University of the Basque Country (UPV/EHU), Vitoria-Gasteiz, Spain; 3 Biomedical Research Networking Centre in Bioengineering, Biomaterials and Nanomedicine (CIBER-BBN), Instituto de Salud Carlos III, Madrid, Spain; 4 Aragón Institute of Engineering Research (I3A), University of Zaragoza, Zaragoza, Spain; 5 Applied Mechanics and Bioengineering Group (AMB), University of Zaragoza, Zaragoza, Spain; 6 Department of Applied Physics, University of Extremadura, Badajoz, Spain; 7 University Institute for Regenerative Medicine and Oral Implantology - UIRMI (UPV/EHU-Fundación Eduardo Anitua), Vitoria, Spain; 8 Singapore Eye Research Institute, The Academia, 20 College Road, Discovery Tower, Singapore

**Keywords:** Molecular conformation, Drug delivery

## Abstract

Cell-laden hydrogel microspheres have shown encouraging outcomes in the fields of drug delivery, tissue engineering or regenerative medicine. Beyond the classical single coating with polycations, many other different coating designs have been reported with the aim of improving mechanical properties and *in vivo* performance of the microspheres. Among the most common strategies are the inclusion of additional polycation coatings and the covalent bonding of the semi-permeable membranes with biocompatible crosslinkers such as genipin. However, it remains challenging to characterize the effects of the interactions between the polycations and the hydrogel microspheres over time *in vitro*. Here we use a force spectroscopy-based simultaneous topographical and mechanical characterization to study polymer-to-polymer interactions in alginate microspheres with different coating designs, maintaining the hydrogels in liquid. In addition to classical topography parameters, we explored, for the first time, the evolution of peak/valley features along the z axis via thresholding analysis and the cross-correlation between topography and stiffness profiles with resolution down to tens of nanometers. Thus, we demonstrated the importance of genipin crosslinking to avoid membrane detachment in alginate microspheres with double polycation coatings. Overall, this methodology could improve hydrogel design rationale and expedite *in vitro* characterization, therefore facilitating clinical translation of hydrogel-based technologies.

## Introduction

Hydrogel-based nano-, micro- and macro-technologies are critical to biomedical research and have an increasing potential to advance clinical medicine through applications including tissue engineering^1,2^, regenerative medicine^3^, drug delivery^4^, biosensors^5^, bioinks^6^, vaccines^7^, and encapsulation for cell-based therapies^8–10^. Among these biotechnologies, the immobilization of therapeutic cells within polymeric matrices coated with semi-permeable membranes has demonstrated encouraging clinical results in the treatment of diverse pathologies so far^11–13^. After four decades of research, alginate-polycation-alginate (APA, either using poly-L-Lysine or poly-L-Ornithine) microsphere remains the design of choice. However, among the limitations of this microsphere type, the lack of long-term stability and poor mechanical properties ultimately lead to compromised biosafety *in vivo*^14,15^.

To address these issues, microsphere performance has been enhanced using additional polycation coatings and/or covalent bonding in the semi-permeable membranes^16^. In particular, genipin, a naturally occurring water-soluble compound, is becoming a popular crosslinker to reinforce the stability of microsphere membranes during and after implantation, partly because of its minimal toxicity and ease of implementation in microsphere fabrication^14,17,18^. Additionally, studies suggest that covalent anchorage between polycation chains with genipin may also increase the overall biocompatibility of APA microspheres when implanted *in vivo*^16^. For these reasons, the introduction of more complex and sophisticated coating designs requires the thorough study of microsphere surface and its implication in the overall refinement of this technology.

Currently, the influence of the chemical composition of alginates on foreign body response, both qualitatively and quantitatively speaking, remains a focus in the field^19^. On the contrary, less attention has been paid to polymer-to-polymer interactions in the surface of the microsphere, and their effect on the final success of the implant. For example, there is a strong correlation between the binding efficiency of polycation chains to alginate matrix and the resulting *in vivo* biocompatibility^15^. Specifically, it has been reported that unbound and exposed polycation leads to more immunogenic membranes, which is of the leading causes of implant failure^15,20,21^. Therefore, to ultimately refine the biosystem design for clinical practice, there is an urgent need for techniques to help unravel the mechanisms leading to implant failure.

In this context, atomic force microscopy (AFM) is a powerful tool to characterize surface topography and can be a proxy to characterize also intermolecular interactions. Moreover, AFM’s sub-nanometric resolution and abilities to work in osmotically relevant liquid environments makes it ideal for the characterization of hydrogels^22,23^. Because sample preparation for AFM does not require drying, fixation, over-crosslinking or contrast, damage to the structure or pore size of hydrogels is minimized^24,25^. Despite the clear advantages of AFM for microsphere characterization, there has been little work on assessing the topography of microspheres in liquid, given the reported complexities of characterizing the topography of hydrogel-based spherical substrates in a liquid environment^26,27^. In fact, AFM can be a difficult technique, which requires a precise calibration standard, and is low-throughput^28^. However, the benefits of characterizing the topography and mechanical properties of hydrogels make this technique unique and worthwhile^29^. Particularly, AFM can help fine-tune microsphere composition, determine the heterogeneity between batches and infer microsphere chances of biocompatibility^30,31^.

In this study, we used force spectroscopy-based AFM imaging to characterize surface properties (i.e., topography and stiffness)^32,33^ of microspheres with different coating designs, including our recently developed genipin-crosslinked double poly-L-Lysine (GDP) membranes^34^. In addition to standard topographical parameters, we propose, for the first time, assessment of peak/valley features in the Z axis through a thresholding analysis, as well as the correlation of simultaneously acquired topographical and mechanical maps of the microsphere surface. Our results provide detailed information about alginate matrix/polycation interactions and reveal the importance of genipin-mediated anchorage to avoid membrane detachment and exposure of immunogenic groups in microspheres with double coatings.

## Results and Discussion

**Figure 1:**
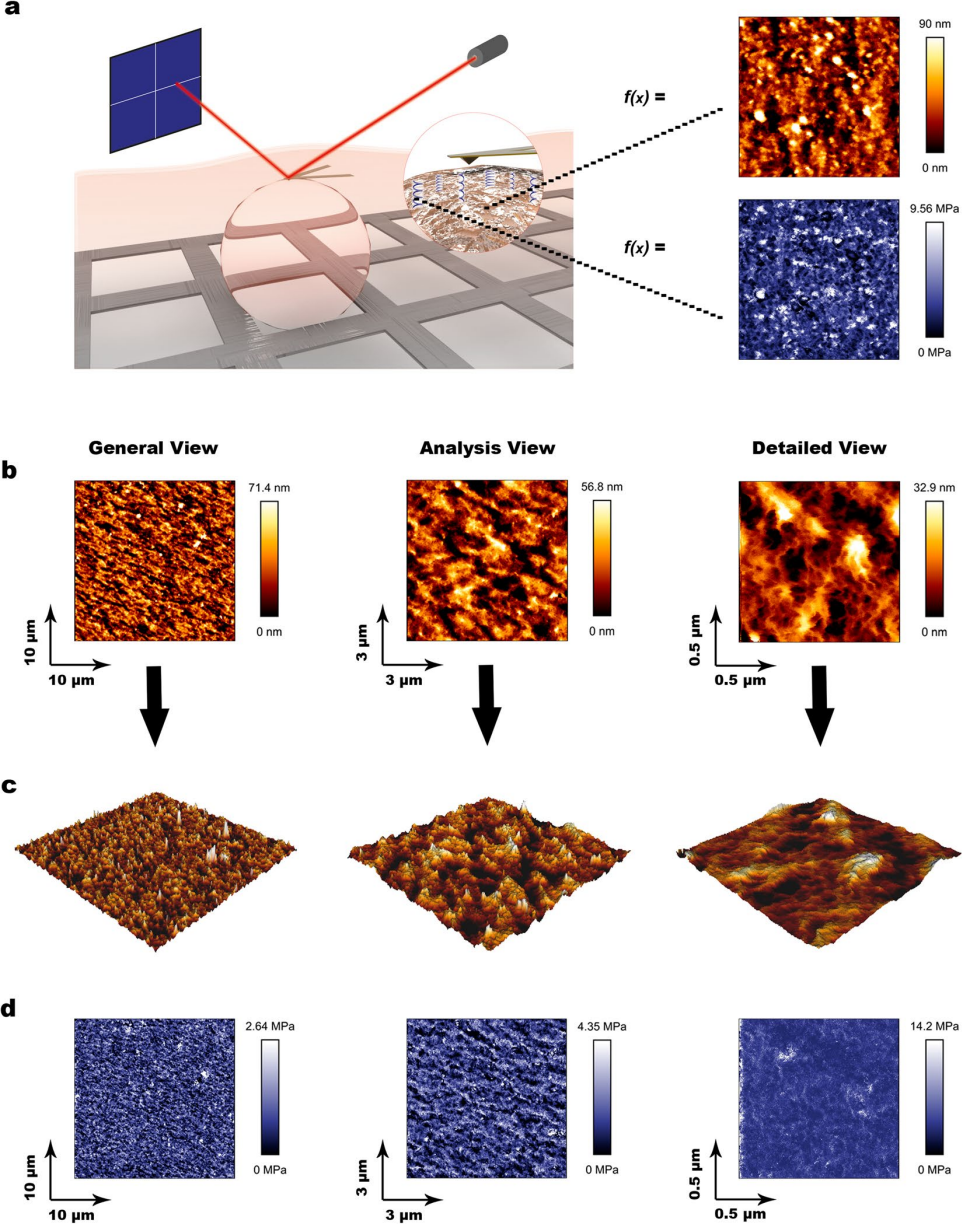
Description of the AFM methodology and representative images of the acquired topography and stiffness maps. (**a)** Schematic depiction of the setup employed to immobilize the alginate microspheres (left), and example of resulting maps for both topography and stiffness (right) measurements. A nylon mesh immobilized the microsphere allowing for stable measurements in liquid. The inset illustrates the indentation of the cantilever on top of the microsphere to acquire force spectroscopy measurements. Resulting data from force spectroscopy was then represented as topography maps (gold) and Young’s moduli were calculated and represented as stiffness maps (blue). (**b)** Representative topography images from alginate microspheres. Three sizes of maps were acquired per microsphere, a 100 μm^2^ general view (left), a 9 μm^2^ analysis view (center), and a 0.25 μm^2^ detailed view (right). The scales are optimized to provide optimal contrast to each image. Notably, optimal scale ranges decrease with higher magnification. (**c)** 3D representations of the topography maps presented in (**b**). (**d)** Stiffness maps acquired simultaneously to the maps presented in (**b**).

We performed our force spectroscopy-based AFM measurements in liquid (serum-free DMEM) in a Petri Dish at 37 °C. A nylon mesh placed in the petri dish immobilized the microspheres without any dehydration step or additional treatment that may modify the structure and properties of the hydrogel (Fig. 1a, left, Fig. S1). Simultaneous collection of topography (gold) and stiffness (blue) values was performed through nanoindentation measurements (i.e., a series of force-spectroscopy measurements performed in a grid shape) (Fig. 1a, right). This setup allowed for three different size square images. All topography images are represented as a heat map (Fig. 1b) that can be 3D rendered (Fig. 1c). The General view (100 μm^2^) provides an overall impression of the regularity of the surface; whereas the Analysis view (9 μm^2^) provides an optimal balance of sampling and resolution of the surface. Finally, the Detailed view (0.25 μm^2^) provides single-nanometer resolution and allows for the investigation of the valleys and smaller features on the microsphere surface. To our knowledge, this is the first time this level of resolution in topography has been reported for alginate microspheres in a liquid environment (representative images in Fig. S2).

Besides, we calculated Young’s moduli (i.e., stiffness) from a Hertz model fitting of the force-spectroscopy measurements. Due to the capabilities of the AFM to characterize surfaces down to hundreds of nanometers into a material, the resulting heatmap (Fig. 1d) provides not only an overall measurement of surface stiffness, but also the spatial distribution stiffness at each point of the capsule topography.

**Figure 2:**
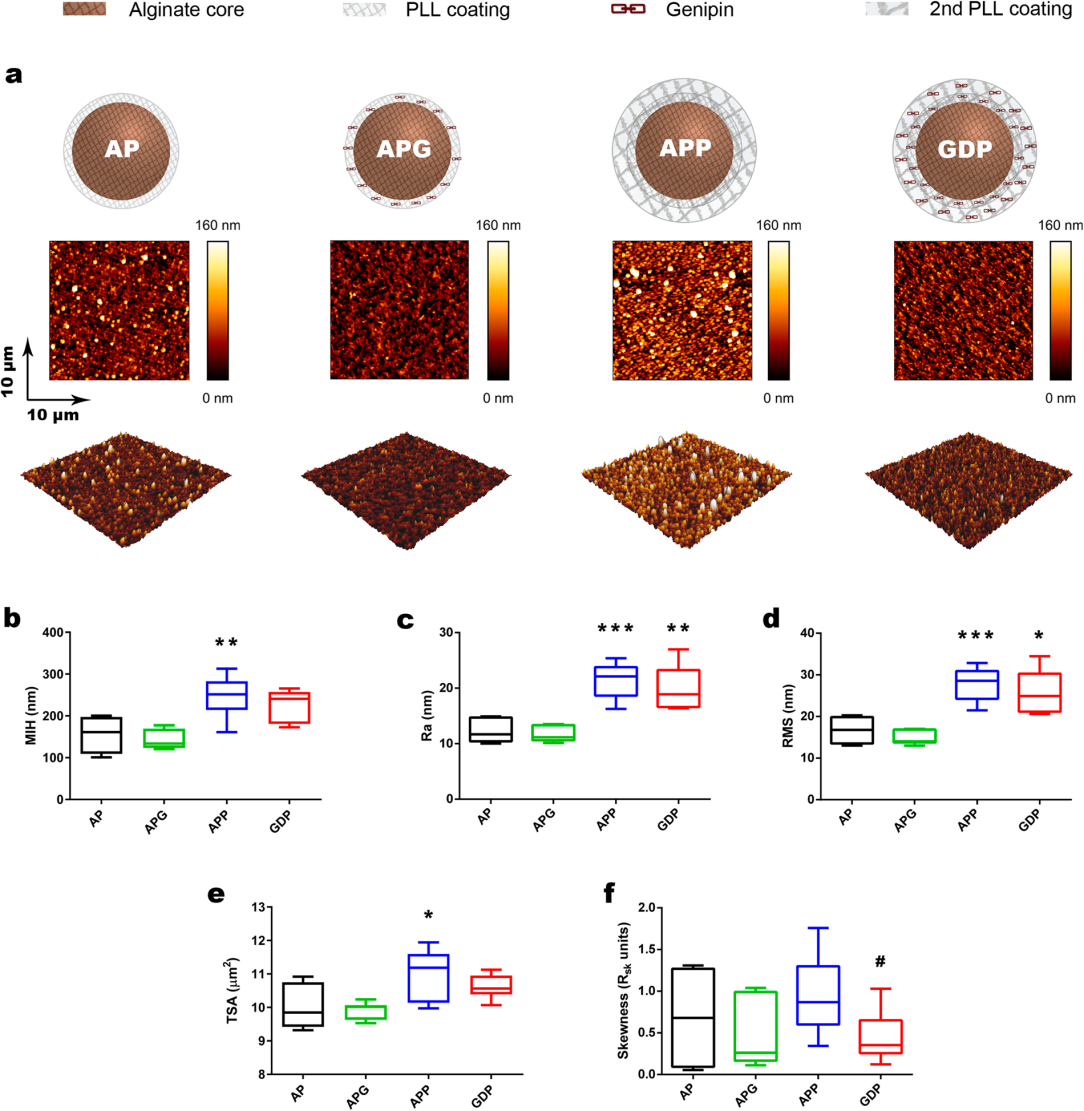
Topographical study of four different coating designs characterized in this paper. (**a)** Schematic depiction showing the different alginate microspheres assayed in the study (top), representative general view 10 × 10 μm topography image for each of the groups (middle) and 3D reconstruction of the topography images shown (bottom). Standard topography parameters were calculated for the alginate microspheres. (**b)** Maximum image height, (**c)** Ra, (**d)** RMS, (**e)** surface area, (**f)** skewness. n = at least 4 samples per group. Boxes depict interquartile range; whiskers depict maxima and minima. One-way ANOVA with Bonferroni post-hoc test was utilized for all topography parameters except for Skewness, after samples passed normality test. Unpaired, two-tailed t-tests was used for Skewness (APP-GDP comparison). *p < 0.05; **p < 0.01; ***p < 0.001 in comparisons against the AP group; ^#^p < 0.05 for APP-GDP comparisons.

Using the experimental setup described above, we studied the effect of double poly-L-Lysine (PLL) coatings and genipin crosslinking on the surface properties of microspheres. To explore more thoroughly the effect of these components, we omitted the final 0.1% alginate coating typically used in microspheres. We characterized four different microsphere compositions: the classical alginate-PLL formulation (AP); AP crosslinked with genipin (APG), AP with a second PLL coating (APP) and finally, our recently developed genipin-crosslinked double poly-L-Lysine membranes (GDP) (Fig. 2a). We tested the correct incorporation of either single or double PLL coatings by means of zeta potential. As expected, APP and GDP designs reflected significant reduction of the net negative charge compared to AP and APG groups with only one PLL coating (**p = 0.0012 in both cases) (Fig. S3a). Besides, genipin is known for emitting far-red fluorescence upon crosslinking completion, therefore we measured this signal as indicator of the crosslinking degree given in the semi-permeable membranes of the microspheres. Both the APG and the GDP groups showed successfully crosslinked membranes (*p = 0.011 and **p = 0.0049 with respect to AP respectively). Moreover, the GDP design produced 7-fold higher fluorescence intensity than APG, confirming a greater degree of covalent anchorage in this group (**p = 0.0049) (Fig. S3b).

We began characterization of these microspheres with classical topography parameters (Fig. S4): the Maximum image height (MIH), Ra, RMS, Total surface area (TSA) and Skewness^35^. We compared the results of different composition microspheres to classical AP microspheres.

MIH (Fig. 2b) is the maximum distance between the highest and lowest points in the scanned images and is a measurement of the surface amplitude (Fig. S4a). We found that APP (double coating) microspheres produced significantly higher topographies than AP (250 ± 15 nm, 60% increase, **p = 0.0060). Interestingly, the crosslinking of the two PLL layers with genipin (GDP microspheres), produced values of MIH that were not significantly different to AP (43% increase, 220 ± 18 nm), suggesting that genipin has a role in flattening microsphere surfaces.

We then measured the changes in Ra along the different microsphere surfaces (Fig. 2c). Ra is a measurement of roughness, calculated as the distance that the surface of interest deviates from a flat plane (parameter illustration in Fig. S4b). We found that Ra values of APP and GDP were significantly higher than those of AP. Interestingly, APP showed a 70% increase in Ra (***p < 0.0001), and GDP showed a 61% increase in Ra as compared to AP control (**p = 0.001), indicating that only APP and GDP (double coating microspheres) are more rough than AP control. Ra values are usually calculated together with other roughness parameters, such as RMS (Fig. 2d). Unlike Ra, RMS calculates the deviations as the area under the curve defined by the surface and a flat plane (Fig. S4b). When we investigated this parameter, results were analogous to those of Ra.

TSA also gives an idea of the overall roughness of the surface (Fig. S4c). The higher the values of TSA, the higher the overall roughness of the surface. Again, the values for APP (Fig. 2e) (11 ± 0.22 μm^2^) alone presented a significant 9.1% increase as compared to AP (10 ± 0.31 μm^2^) (*p = 0.044). Conversely, no significant differences were found between AP, APG, and GDP.

Finally, we assessed Skewness, which indicates the symmetry (or lack thereof) in the distribution of the overall height of the surface using half the MIH as reference (Fig. 2f). In other words, it indicates the regularity of a surface (e.g., the presence of a few high peaks in an overall flat topography would result in high Skewness values) (Fig. S4d). For skewness, we observed a significant difference between APP and GDP (#p = 0.026), indicating again the strong influence of the genipin crosslinking in the microsphere topography after the second PLL coating. This indicates a lower predominance of peaks in GDP, and therefore the generation of more homogeneous and symmetric surfaces than in APP microspheres.

**Figure 3:**
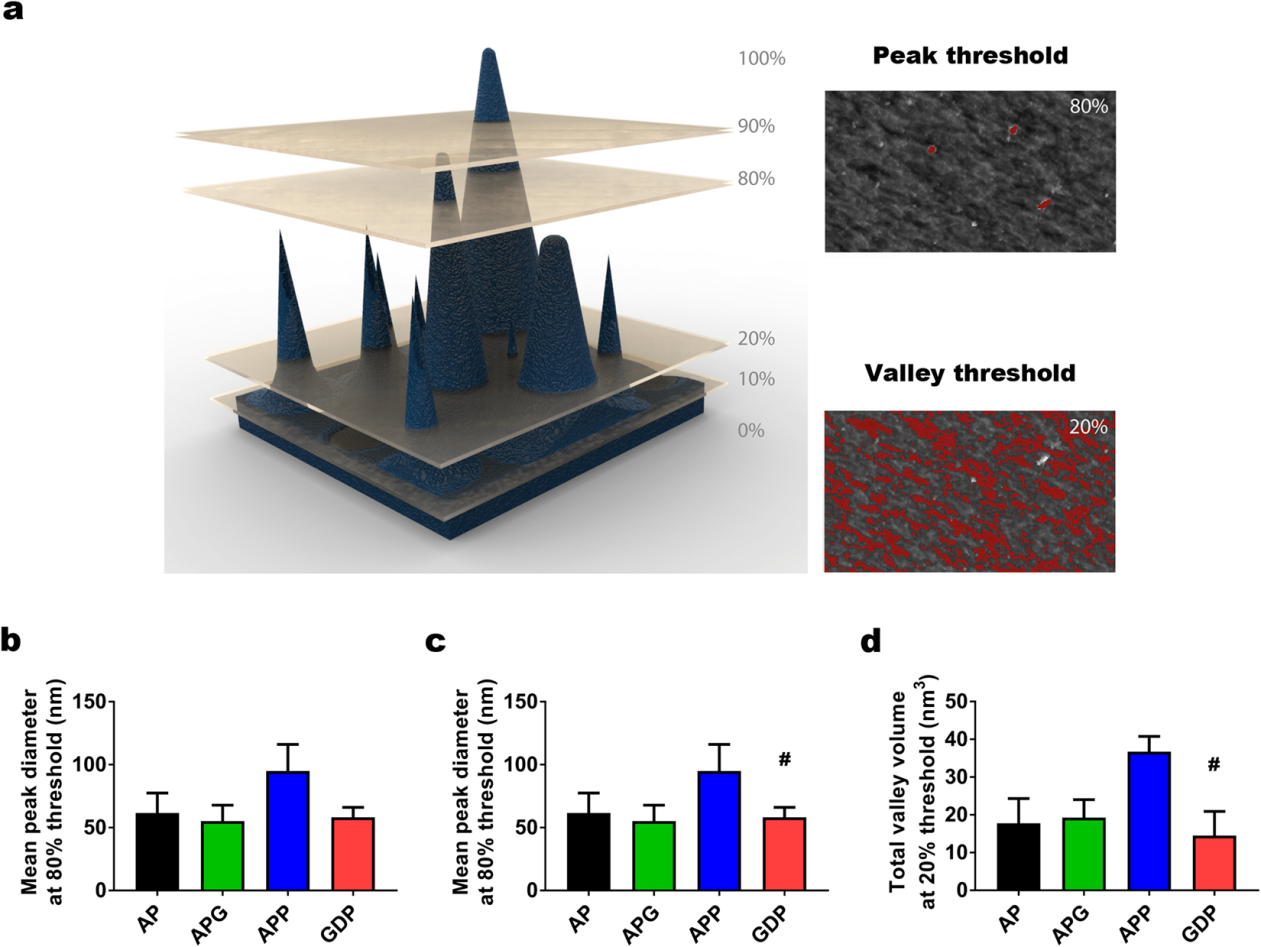
Results of the thresholding analysis for the different alginate microspheres. (**a)** A schematic representation of a topography example (left, dark blue) is sectioned (planes depicted in gold) to illustrate thresholding analysis at 10, 20 (for valley analysis), 80 and 90% (for peak analysis) of the maximum image height (MIH). The result of these sections is depicted for a representative image on the right. The areas corresponding to peaks above 80% of the MIH are highlighted in red, as are the areas corresponding to valleys below 20% of the MIH. (**b)** Mean peak diameter calculated through grain analysis at 80% threshold of the MIH. (**c)** Mean valley diameter calculated through grain analysis at threshold 20% of the MIH. Xy scale of the thresholded image is 3 × 3 µm, whereas z scale is 74 nm. (**d)** Total valley volume in absolute value, calculated through grain analysis similarly to (**c**). n = at least 5 samples per group. All graphs depict mean ± standard error of the mean. One-way ANOVA with Bonferroni post-hoc test. ^#^p < 0.05 for APP-GDP comparisons.

To further characterize the influence of both double coatings and genipin on microsphere surface properties, we next performed a thresholding analysis on our topographies. This analysis consists of choosing different image heights and generating a section of the image at the chosen height. Typically, these heights are represented as a percentage of the MIH (Fig. 3a).

Following this procedure, we defined a threshold to study the peaks of our topographies^36^, located at 80% of the MIH, (Fig. 3a, *peak threshold*). On the images subjected to this threshold, we performed a grain analysis and studied the mean peak diameter for each microsphere composition (Fig. 3b). We found no significant differences in any of the comparisons.

We performed a similar threshold for valleys, at 20% of the MIH (Fig. 3a, *valley threshold*). From this threshold, we first quantified the mean valley diameter (Fig. 3c). We observed that GDP showed a 35% reduction in mean valley diameter (48 ± 9.2 nm) which was significantly smaller than APP (#p = 0.039).

Finally, with this threshold we studied the total valley volume (Fig. 3d), defined as the volume comprised by the 20% threshold plane and the topography below. This parameter provides insight into the possible presence of pores in the surface of the microsphere. Results were analogous to those of mean valley diameter (#p = 0.034). Overall, our results indicate that the topography of GDP microspheres is more similar to AP and APG than it is to APP. However, this analysis did not fully reflect the differences in topography that we were observing. Therefore, we decided to perform a more in-depth analysis of peak-valley features on our topography images.

**Figure 4:**
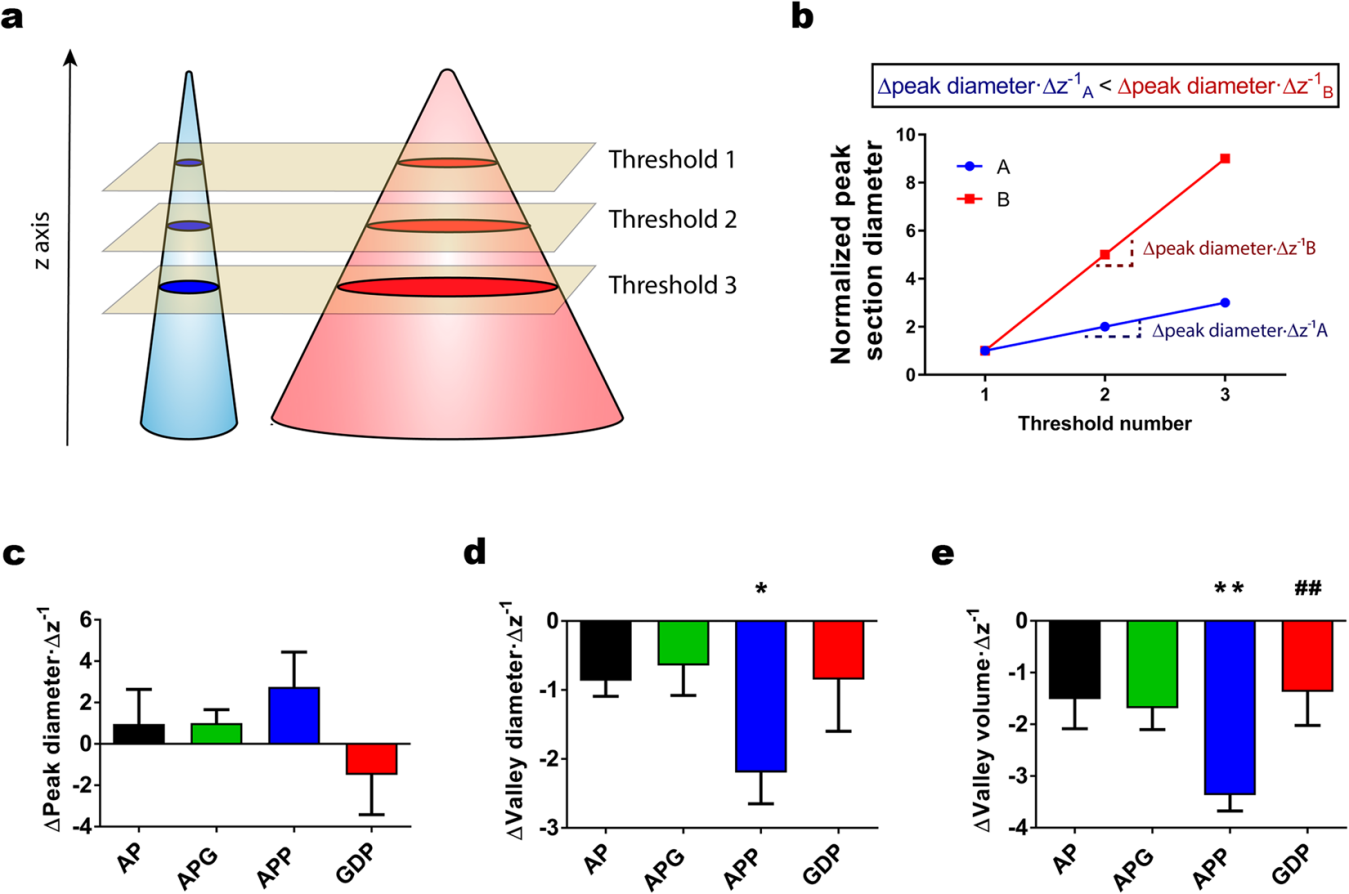
Results of the thresholding analysis along the z axis for the different alginate microspheres. (**a)** Schematic of the sections resulting from thresholding at different heights in two different peak models. (**b)** Model graph of the peak diameter for the thresholds performed along the z axis on the model peaks shown in (**a**). This graph also illustrates the parameter Δpeak diameter · Δz^−1^ used in subsequent panels and its variation for the two model situations described in (**a**). (**c)** Δpeak diameter · Δz^−1^ calculated from a line fitting of the 80, 85 and 90% thresholds shown in Fig. S5a for the different microsphere groups. (**d)** Δvalley diameter · Δz^−1^ calculated from a line fitting of the 20, 15 and 10% thresholds, as shown in Fig. S5b. (**e)** Δvalley volume · Δz^−1^ calculated similarly to (**d**), from the line fittings shown in Fig. S5c. n = at least 5 samples per group. All graphs depict mean ± standard error of the mean. Unpaired, two-tailed t-test. *p < 0.05; **p < 0.01 for AP-APP comparisons; ^##^p < 0.01 for APP-GDP comparisons.

To further characterize the shape and size of the peaks generated on the surface of the microspheres by the different compositions, we defined thresholds along the Z axis of the surface at 80, 85 and 90% of the MIH for the peaks, and we performed a grain analysis in these three thresholds (Fig. 4a). Qualitatively, the profiles of APG and GDP resemble those of AP more than those of APP. Then, we performed a line fitting of these points to observe and compare the evolution of the mean peak size along the z axis (∆Peak area·∆z^−1^) (Figs. 4b and S5). However, we did not find any statistically significant results in ∆Peak area·∆z^−1^ (Figs. 4c and S5a).

Next, we defined similar thresholds to study valleys (20, 15 and 10% of MIH). With this, we studied the ∆Valley diameter·∆z^−1^. In this case, APP produced the steepest curves, whereas the curves for AP, APG and GDP were mostly flat (Fig. S5b). When we studied the slopes of the line fittings, we observed that the mean valley slope of APP topographies (−2.2 ± 0,46) was 250% more negative than that of AP (−0.86 ± 0.23), which was statistically significant (*p = 0.020) (Fig. 4d).

We performed the same analysis on the total valley volume, which produced similar results to the mean valley diameter. APP produced significantly steeper transitions (∆Valley volume·∆z^−1^) among the three examined thresholds (Fig. S5c). For ∆Valley volume·∆z^−1^, APP values were significantly more negative than both AP (**p = 0.0084) and GDP (##p = 0.0086) (Fig. 4e).

Overall, these results indicate that APP presents different topographies from AP and APG (single coating microsphere compositions). In particular, peaks in APP get thicker as we move closer to the lowest point in the topography. In the case of valleys, APP presented steeper valleys, initially bigger in diameter but quickly narrowing as we move closer to the lowest point in the topography. The resemblance of GDP and AP surfaces contrasts with their different composition (i.e., GDP microspheres have two PLL coatings), which suggests that genipin crosslinking normalizes the topographies that the second PLL coating generates.

**Figure 5:**
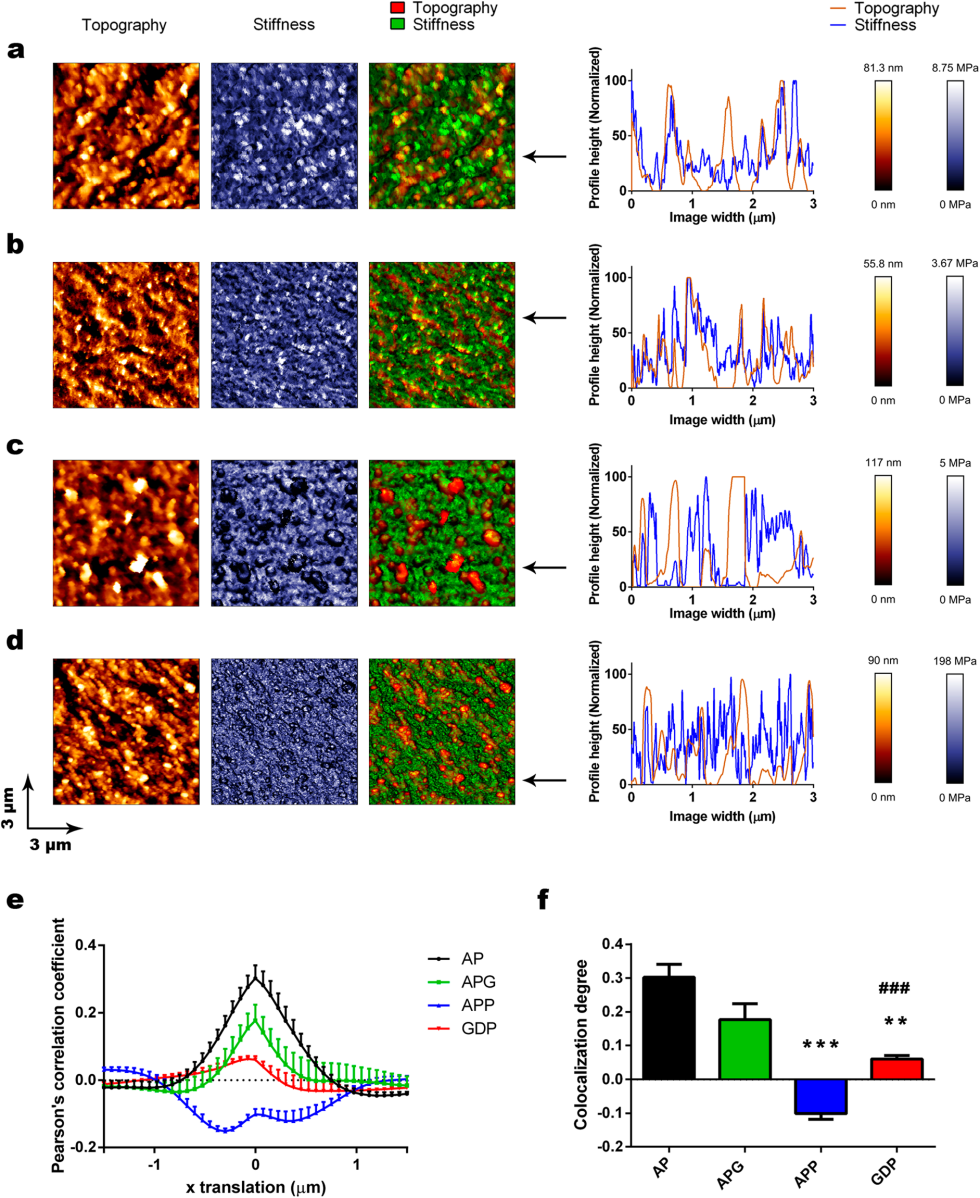
Colocalization of topography and stiffness maps of the microspheres. Sample topography (gold), stiffness (blue) and topography (red)- stiffness (green) overlays are shown for microsphere types (**a)** AP, (**b)** APG, (**c)** APP, (**d)** GDP. Line profiles of topography (gold) and stiffness (blue) are shown on the right of each of the maps. (**e)** Colocalization graphs for topography and stiffness are shown as a function of the Pearson correlation coefficient graphed as a function of x translation. One-sample t-tests were used to determine if the central points were significantly different from 0. (**f)** Colocalization degrees calculated from (**e**) are depicted for each of the microsphere types. n = at least 6 samples per group. All graphs depict mean ± standard error of the mean. Data was proved normal and then subjected to ANOVA and Tamhane post-hoc test. **p < 0.01; ***p < 0.001 in comparisons against the AP group; ^###^p < 0.001 for APP-GDP comparisons.

Polymer-to-polymer interactions between PLL chains and alginate matrix are critical to avoid membrane detachment and exposure of immunogenic positive groups, which ultimately leads to graft failure. To explore these interactions in detail, we propose a novel procedure based on overlapping of topography (gold) and stiffness (blue) maps (Fig. 5a–d) and subsequent cross-correlation analysis according to Van Steensel *et al*. (Fig. 5e) (detailed explanation of analysis and interpretation in Material and Method section)^37^. Thus, both the AP and APG groups (one coating) showed numerous co-localizing points (Fig. 5a,b; yellow spots), matching line profiles and highly positive cross-correlation curves (Fig. 5e), which indicates strongly correlated topography and stiffness maps. Conversely, the APP group (double coating) exhibited peaks in topography corresponding to valleys in stiffness and vice versa (Fig. 5c). In addition, spatial cross-correlation analysis resulted in a negative function (Fig. 5e). All these data point to inversely correlated maps in APP. Strikingly, when we used genipin to crosslink PLL membranes in GDP microspheres (double coating) anti-colocalizing both spots and line profiles disappeared in overlapping maps (Fig. 5d). Indeed, genipin was able to restore the positive values in cross-correlation analysis, obtaining again a weak correlation curve more similar to those obtained for AP and APG groups (Fig. 5e).

We then focused on Pearson’s correlation coefficients obtained at central points of the cross-correlation curves (0 shift in the *x* axis) to compare colocalization degrees among groups. As a result, APG showed non-significant differences as compared to AP group (both single coating), while the negative values of APP group (double coating) did exhibit statistically significant differences (***p = 0.00006) (Fig. 5f). On the contrary, the positive values are restored in GDP group and produced statistically significant differences when compared to APP (###p = 0.00013), although Pearson’s coefficient was significantly lower than those of AP and APG (**p = 0.003) (Fig. 5f).

From this data, we can draw important information regarding alginate / PLL interaction in microspheres. Particularly, a strong correlation between topography and stiffness may indicate that interpenetrating PLL membranes are well integrated into the alginate matrix (AP and APG groups). On the contrary, inversely correlated profiles may be indicative of poorly interacting polymers, which would result in detached membranes that are expanded by repulsion forces and appear as “soft peaks” by AFM and posterior cross-correlation analysis (APP group). Finally, the recovery of the Pearson’s coefficient in GDP microspheres from APP, is interpreted by authors as a tighter organization of PLL chains, crosslinked by genipin, which would flatten out the features observed in APP. Therefore, the surface features of GDP are more similar to those of AP and APG, which is in agreement with the results obtained by both standard topographical analyses and thresholding analyses.

**Figure 6:**
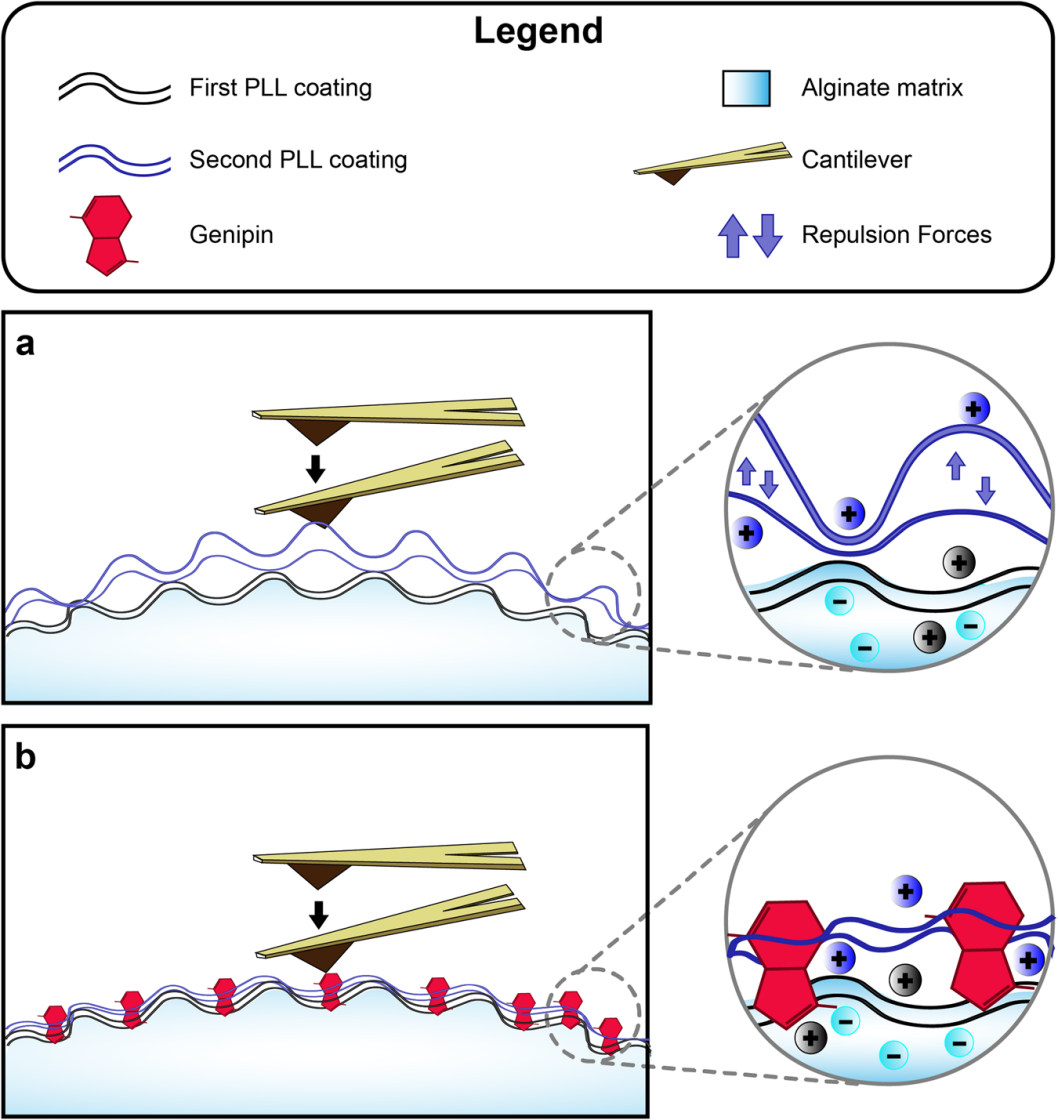
Proposed model of the interactions between PLL and alginate in function of genipin crosslinking for the studied microspheres. The first PLL coating is well integrated in the alginate matrix by means of attractive electrostatic interactions. The second PLL coating interacts in a different way depending on whether or not genipin is included. (**a**) In the APP microspheres, the PLL chains of the second coating find less available negative charges in the alginate matrix to interact with. In addition, the positive charges of the first coating produce repulsion forces that hamper the integration of the second coating. This results in a loosely attached second PLL layer. The second PLL layer is therefore bulkier and generates high peaks on the surface that are detected as soft by AFM. (**b**) In GDP microspheres, genipin creates covalent bondings that pin both PLL coatings strongly to the alginate matrix, creating more densely packed membranes. Genipin crosslinking is enough to overcome the repulsion forces between the PLL chains, making possible for the second coating to reach the alginate matrix and increase the PLL-alginate interactions. These features can be readily probed with AFM, resulting in the disappearance of the above-mentioned soft peaks.

In light of all the evidence presented in this paper (summarized in Table 1), we hypothesize that interpenetrating PLL membranes in single-coating microspheres are well integrated into the alginate matrix (AP and APG groups) (Fig. 6, black lines). At this level, genipin crosslinking does not affect PLL/alginate interactions. However, the addition of a second PLL layer (Fig. 6, blue lines) generated different surface features depending on whether or not genipin was present. On the one hand, double PLL coatings without genipin crosslinking (APP group) generated higher peaks and more pronounced transitions along the z axis. These higher peaks were perceived as soft protuberances by AFM and posterior cross-correlation analysis. Soft peaks may be indicative of a weak interaction between alginate and PLL, suggesting that the second PLL (positively charged) membrane was detached from the surface and expanded as a result of repulsion forces among PLL chains of both coatings (Fig. 6a, inset). On the other hand, the addition of genipin in the second PLL coating (GDP, Fig. 6b) produced more similar features to those of AP and APG, despite having two coatings. These features included more shallow and symmetric peaks, moderate transitions along the z axis, and the disappearance of the soft peaks observed in the APP group. Thus, in GDP, genipin may be anchoring PLL chains of both coatings, thereby compressing and maintaining the whole PLL network in close contact with the alginate matrix. Repulsion forces between PLL chains not integrated in the alginate matrix would be overpowered by the covalent bonds of the genipin crosslinking, thereby reducing the exposure of positive charges (Fig. 6b, inset). This covalent bond-based compression and shielding of charges would explain the resemblance of the surface features of GDP to those of AP and APG, despite having two PLL coatings.

**Table 1:** Summary of the findings of this manuscript for APP and GDP, and interpretation of the effect of genipin in the surface topography of the microsphere.

	APP	GDP	Effect of genipin
MIH	Increased	Similar to AP	Decrease
Ra & RMS	Notably Increased	Increased	Slight decrease
TSA	Increased	Similar to AP	Decrease
Skewness	Similar to AP	Decreased	Lower asymmetry profiles
Peaks	Similar to AP	Similar to AP	No effect
Valleys	Increased	Similar to AP	Normalization
Topography in Z	Changing	Constant	Normalization
Colocalization of Topography/ Stiffness	Inversely colocalized	Non colocalized	Normalization

**Figure 7:**
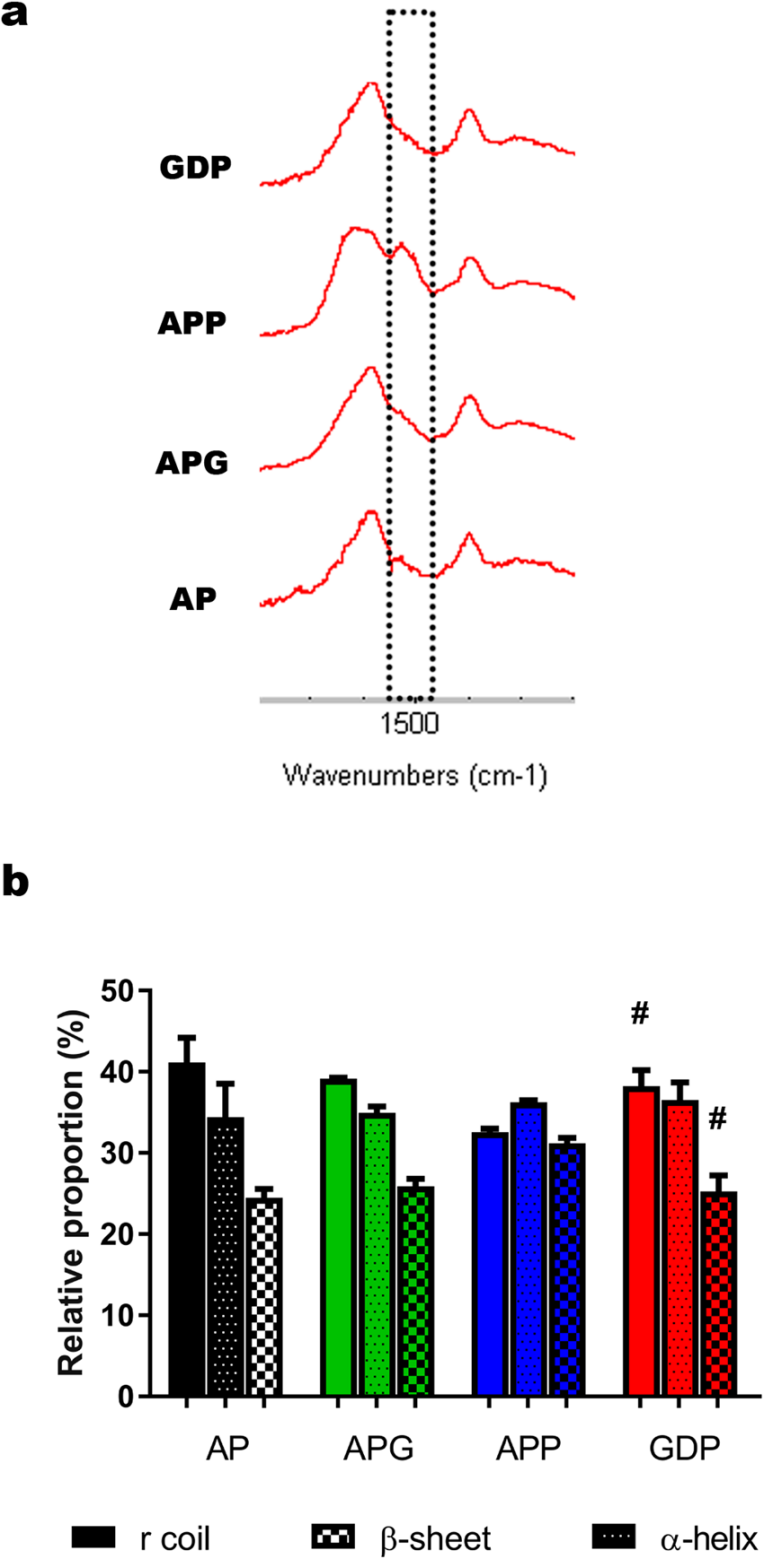
Surface characterization studies. (**a)** Representative ATR-FTIR spectra (1800–1200 cm^−1^) of microspheres showing the Amide II absorption (≈1500 cm^−1^) of PLL (dashed line). (**b)** Analysis of amplified Amide II band from (**a**) to determine PLL conformation. 1555 cm^−1^, Random Coil; 1537 cm^−1^, α-Helix; 1522 cm^−1^, β-sheets. Relative height of each of the three peaks was expressed as the percentage of each PLL conformation. n = 3 samples per group (n = 2 AP and APG). Error bars: mean ± standard error of the mean. Unpaired, two-tailed t-test. ^#^p < 0.05 for APP-GDP comparisons.

To validate this hypothesis, we performed a physicochemical analysis of the composition of microsphere surfaces using infrared spectrophotometry by Fourier transform-attenuated total reflectance (ATR-FTIR). We examined the shoulder corresponding to the Amide II absorbance band of PLL (≈1500 cm^−1^) as an indicator of exposed PLL^15,38^. Thus, spectra of the APP group showed a pronounced shoulder when we added a second PLL coating to the microspheres (Fig. 7a). Interestingly, genipin crosslinking successfully reduced the increased PLL exposure as denoted by the spectra of GDP microspheres. Further analysis of the amplified Amide II band of the PLL, following data interpretation proposed by other authors^39,40^, confirmed significant differences in PLL conformation between GDP and APP microspheres (^#^p < 0.05) (Fig. 7b). Particularly, we observed a significant increase in the proportion of Random Coil conformation (high alginate-PLL interaction) of GDP capsules (#p = 0.045), while α-helix conformation (low alginate-PLL interaction) was reduced (^#^p = 0.046) (Fig. 7b). These results, together with the disappearance of the Amide II shoulder shown in Fig. 7a, support that genipin crosslinking compresses the loose PLL network (high exposure) of APP membranes and sinks them in the alginate matrix.

## Conclusions

In summary, we present a force spectroscopy-based simultaneous topographical and mechanical characterization to study polymer-to-polymer interactions in alginate microspheres with different coating designs. In addition to classical parameters, we also explored topographical changes along the z axis (thresholding analysis) and cross-correlation between topography and stiffness profiles with resolution down to tens of nanometers. From these observations, we demonstrated the importance of genipin crosslinking to avoid membrane detachment and exposure of immunogenic charges in alginate microspheres with double polycation coatings. We believe the methodology presented in this paper may be useful in the development and quality control of new microsphere technologies for cell encapsulation. Particularly, this methodology could complement *in vitro* testing to select hydrogel technologies most likely to perform well *in vivo*. A more combined pre-screening would both reduce the need for experimentation animals and expedite research. We envision that multiple hydrogel-based biotechnologies ranging from drug or cell delivery systems to biosensors could benefit from the approach described in this paper.

## Materials and Methods

### Microsphere preparation

Microsphere preparation was carried out by using an electrostatic droplet generator with slight modifications of the procedure designed by Lim and Sun^41^. Briefly, 1.5% ultra-pure low-viscosity high glucuronic acid alginate (UPLVG) (FMC Biopolymer, Norway) suspension was extruded through a 0.35 mm gauge needle at a 5.9 ml/h flow rate using a peristaltic pump. The drops were gelled in a 55 mM CaCl_2_ (Sigma Aldrich, St. Louis, MO, USA) solution and maintained in a shaker for 15 min to enable all the beads to reach the ion exchange equilibrium. Subsequently, these beads were washed and coated according to the desired microsphere design, as described previously^34^. Briefly:

AP is Classical alginate-PLL (PLL hydrobromide M_w_ 15–30 kDa) (Sigma Aldrich, St. Louis, MO, USA) microspheres; APP is made from AP microspheres with a second coating of PLL, APG is generated by crosslinking AP microspheres with genipin (Wako Chemicals GmbH, Germany) and GDP consists of APG microspheres with a second coating of PLL, then subjected to a second crosslinking reaction.

PLL and genipin were used as 0.05% and 0.1% solutions respectively, both in DPBS with calcium and magnesium (Thermo Fisher Scientific, MA, USA). All coating and crosslinking steps were carried out by maintaining the microspheres 5 mins in suspension with the corresponding solution, followed by a washing step in a DPBS solution with calcium and magnesium. The typical second 0.1% alginate coating was omitted in order to explore more thoroughly the effect of PLL and genipin. The whole process was performed at room temperature and under aseptic conditions. Resulting particles were stored in a flask filled with complete medium at 37 °C in a standard incubator with 5% CO_2_/95% air atmosphere.

### Zeta potential measurements

Microcapsules were removed from their storage medium and carefully washed in KCl 10^−3^ M. Then, 2 ml of microcapsules were transferred to a syringe for zeta potential determination.

Experiments were carried out with an Electrokinetic Analyzer (EKA, Anton Paar, Austria) using a cylindrical cell with the option of streaming potential (V_str_) and 600 mbar of ramp pressure (p). The slope of the plot (dV_str_/dp) was directly related to zeta potential (ζ):

$$\zeta ={{\rm{K}}}_{{\rm{cell}}}\frac{{\rm{\eta }}}{{{\rm{\varepsilon }}}_{{\rm{r}}}{{\rm{\varepsilon }}}_{{\rm{o}}}}\frac{{{\rm{dV}}}_{{\rm{str}}}}{{\rm{dp}}}$$

η and ε_r_ being the viscosity and the relative permittivity of the electrolyte, ε_0_ the vacuum electric permittivity and K_cell_ is the conductivity of the flow path^42^.

### AFM measurements

Mechanical characterization of microspheres was measured by AFM on a JPK Nanowizard 3 (JPK instruments, Germany) coupled with an inverted microscope (NIKON Ti, Nikon Instruments) keeping a constant temperature through a Petri Dish Heater (JPK instruments, Germany).

### Tip calibration

PNP- TR cantilevers (NanoAndMore, Switzerland) were chosen due to their quadratic pyramid shape, which could be accounted for in later Young’s Moduli analyses. Particularly, of both tips present in the cantilever, tip A was chosen due to the adequacy of its nominal spring constant (0.32 N·m^−1^) for stiffer microsphere samples due to crosslinking. The spring constant was precisely characterized for each tip by Thermal Noise Method prior to a new set of measurements.

### Microsphere sample preparation

Young’s moduli calculation and topography imaging were performed using QI Mode as previously described^43^. Briefly, a nylon mesh with 330 µm openings (Labopolis S.L., Spain) was immobilized on a Petri dish (TPP Techno Plastic Products AG, 93040) using a standard 2-component cyanoacrylate adhesive. Once dry, the dish was filled with serum-free media, while avoiding bubble lodging in the mesh, and let to reach 37 °C in a PetriDish Heater. Then, an aliquot of microsphere suspension was collected in sterile conditions. The microspheres were carefully resuspended with a specialty wide orifice tip (Sigma-Aldrich, P6800), added on top of the Petri dish and let to sediment and accommodate in the mesh openings for 5 min.

### QI imaging

A microsphere centered within one of the openings of the nylon mesh was identified through the inverted optical microscope. The calibrated cantilever was positioned on top of the microsphere to carry out the AFM force spectroscopy-based measurements. Initially, high Z-lengths and slow speeds (10 µm·s^−1^) were used to maximize the signal-to-noise ratio. Subsequently, speeds up to 120 µm·s^−1^ were used, the piezo range was reduced to 5 µm to increase resolution, and Z-length was shortened to the nm scale in order to minimize sample drift effects during the acquisition, but programmed to at least double the image height. Final image resolution was 256 × 256 pixels. At least three microspheres were investigated per type, and at least five square images (9 µm^2^, Analysis view) were extracted for the statistical characterization. General overview images (100 µm^2^) and detailed view images (0.25 µm^2^) were also taken to assess the general aspect of the microsphere surface and details of the peaks and valleys.

### Image processing

Images were processed with DP software (JPK Instruments, Germany). Raw images were first flattened by means of a second order polynomial line fit, which accounts for the overall curvature of the spherical sample. Furthermore, images were subjected to a low degree smoothing, to render pixel transition less evident. Finally, a gold color scale was applied to all topography images.

### Data processing

#### Bidimensional statistics

Bidimensional statistics were performed on height topography images using Gwyddion open-source software^44^. Particularly, bidimensional parameters of maximum image height (MIH), Ra, RMS, Skewness and total surface area (TSA) were studied for the purposes of this paper. Furthermore, different height thresholds were applied to height maps in order to determine and quantify grain (i.e., peak and valley) prevalence, similarly to those previously described by Arndt *et al*.^36^. Specifically, thresholds were applied at 10, 15 and 20% for valley determination and 80, 85 and 90% for peak diameter quantification. Grain statistics were applied on the resulting thresholded images. We particularly chose mean peak diameter, mean valley diameter and total valley volume as parameters to characterize the grains.

#### Young’s Moduli extraction

Young’s Moduli were extracted from nanoindentation curves comprised in the QI images. For the analysis, data were processed with DP Software (JPK Instruments, Germany) and fitted to a Hertz model modified for quadratic pyramid indentors^24,45^. Poisson ratio was set to 0.5. Calculated values were represented in a map form, and a blue color scale was applied to all Young’s Moduli images. A representative force curve with the Hertz model fitting and a representative histogram from a whole image are presented in Fig. S6.

#### Correlation analysis of topographical and stiffness images

Colocalization studies of topography (gold) and stiffness (blue) maps were performed in ImageJ^46^. Firstly, images were converted into 16-bit binary format. For a clear representation of colocalizing features, topography images were depicted in red and topography maps in green. Separated color images were merged to find yellow colocalizing spots. Sample line profile plots were also extracted from the above mentioned colocalization maps for a more detailed view of colocalization between both maps.

The cross-correlation analysis was performed in ImageJ following the procedure described previously by Van Steensel *et al*.^37^. Briefly, in overlapped topography and stiffness maps, the topography map was shifted over a particular distance Δ*x* voxels in the *x*-direction to the right (positive values) and to the left (negative values). For each shift, Pearson moment-product correlation *r*ρ was calculated and plotted against Δ*x*. This method ensures that *r*ρ values are not dependent on the relative intensities of each map or on the settings of the microscope for each image^37^. In addition, pixels with higher signal (or height) within the image, contribute more strongly to the *r*ρ value, which allow removing great part of the image background contribution. Average correlation curves + SEM were generated from at least 6 pairs of topography/stiffness images. To determine if the spatial Pearson moment-product correlation for each group was significantly different from 0 in the central point, one-sample t-tests were used.

### ATR-FTIR spectra

Before being analyzed, washed and pelleted microspheres (1 mL) were dried for a minimum of 24 h in a sterile laminar flow hood, followed by a second drying of at least 24 h more in a vacuum. Attenuated total reflectance Fourier transform infrared (ATR-FTIR) spectra were recorded on a Nicolet iS10 spectrometer equipped with a Thermo Scientific Smart iTR-ATR accessory using 32 scans with a resolution of 4 cm^−1^ in 4000–400 cm^−1^ region. A bare crystal background was used as a blank for all spectra collected. For each microsphere composition, 3 independent samples were examined.

The shoulder corresponding to the Amide II absorbance band of PLL (≈1540 cm^−1^) was examined as indicator of the exposed PLL^15,38^. PLL-alginate interaction was further analyzed by exploring in detail the amplified Amide II band of the PLL, which is actually composed of various peaks corresponding to the three possible PLL conformations: 1555 cm^−1^ frequency represents random coil conformation (high interaction), 1537 cm^−1^ α-helix and 1522 cm^−1^ β-sheets (low interaction)^39,40^. Relative height of each peak with respect to the total heights of the three peaks was considered as the percentage of each PLL conformation.

### Statistics

Graphs are presented as mean ± SEM. In box plots, the line within the box shows the median, and the box edges delimitate the interquartile range (Q25-Q75). Whiskers extend to highest and lowest values from the box hinge. Graphs were plotted using Graphpad Prism v7.04 (Graphpad Prism Inc., La Jolla, CA, USA).

All statistical computations were executed using IBM SPSS Statistics 22. Independent two-sample Student’s t-test was used to detect significant differences between two groups, after challenging the normal data distribution by Shapiro Wilks test. One-way ANOVA was used for comparison among multiple groups. For direct one-to-one comparisons, homoscedasticity was first challenged through Levéne’s test. If variance homogeneity was proved, Bonferroni post-hoc test was used for the comparisons. Conversely, if variance homogeneity was disproved, Tamhane’s test was used for the comparisons. Each figure legend specifies the statistical test utilized. Significance level was set at 0.05.

## Supplementary information

Supplementary Information

## Data Availability

Due to the high volumes of data utilized in this manuscript, data will be made available upon request.
