# Supplementary material for: Force spectroscopy-based simultaneous topographical and mechanical characterization to study polymer-to-polymer interactions in coated alginate microspheres
Source: Sci Rep. 2019 Dec 27;9:20112. doi: 10.1038/s41598-019-56547-z (PMC6934587; doi:10.1038/s41598-019-56547-z)
Supplement: Supplementary file 1 — Supplementary Information [file 41598_2019_56547_MOESM1_ESM.docx]

**Force spectroscopy-based simultaneous topographical and mechanical characterization to study polymer-to-polymer interactions in coated alginate microspheres**

**Authors:** Maria Virumbrales-Muñoz^1^*, Edorta Santos-Vizcaino^2,3^*, Laura Paz^4,5,6^, Amparo Maria Gallardo-Moreno^2,7^, Gorka Orive^2,3,8,9^, Rosa Maria Hernandez^2,3^, Maria Luisa Gonzalez-Martin^2,7^, Luis Jose Fernández^4,5,6†^, Jose Luis Pedraz^2,3†^, Ignacio Ochoa^4,5,6†^

**Affiliations**

1. Department of Biomedical Engineering, University of Wisconsin, Madison, WI, USA

2. NanoBioCel Group, Laboratory of Pharmaceutics, School of Pharmacy, University of the Basque Country (UPV/EHU), Vitoria-Gasteiz, Spain

3. Biomedical Research Networking Centre in Bioengineering, Biomaterials and Nanomedicine (CIBER-BBN), Spain

4. Group of Applied Mechanics and Bioengineering (AMB), Centro de Investigación Biomédica en Red. Bioingenieria, biomateriales y nanomedicina (CIBER-BBN), Spain.

5. Aragón Institute of Engineering Research (I3A), University of Zaragoza, Spain.

6. Aragon Institute of Biomedical Research, Instituto de Salud Carlos III, Spain.

7. Department of Applied Physics, University of Extremadura, Badajoz, Spain.

8. University Institute for Regenerative Medicine and Oral Implantology - UIRMI (UPV/EHU-Fundación Eduardo Anitua), Vitoria, Spain.

9. Singapore Eye Research Institute, The Academia, 20 College Road, Discovery Tower, Singapore.

* These two authors contributed equally to this work.

^†^ Luis Jose Fernández, Jose Luis Pedraz and Ignacio Ochoa are corresponding authors

**Supporting information**

**
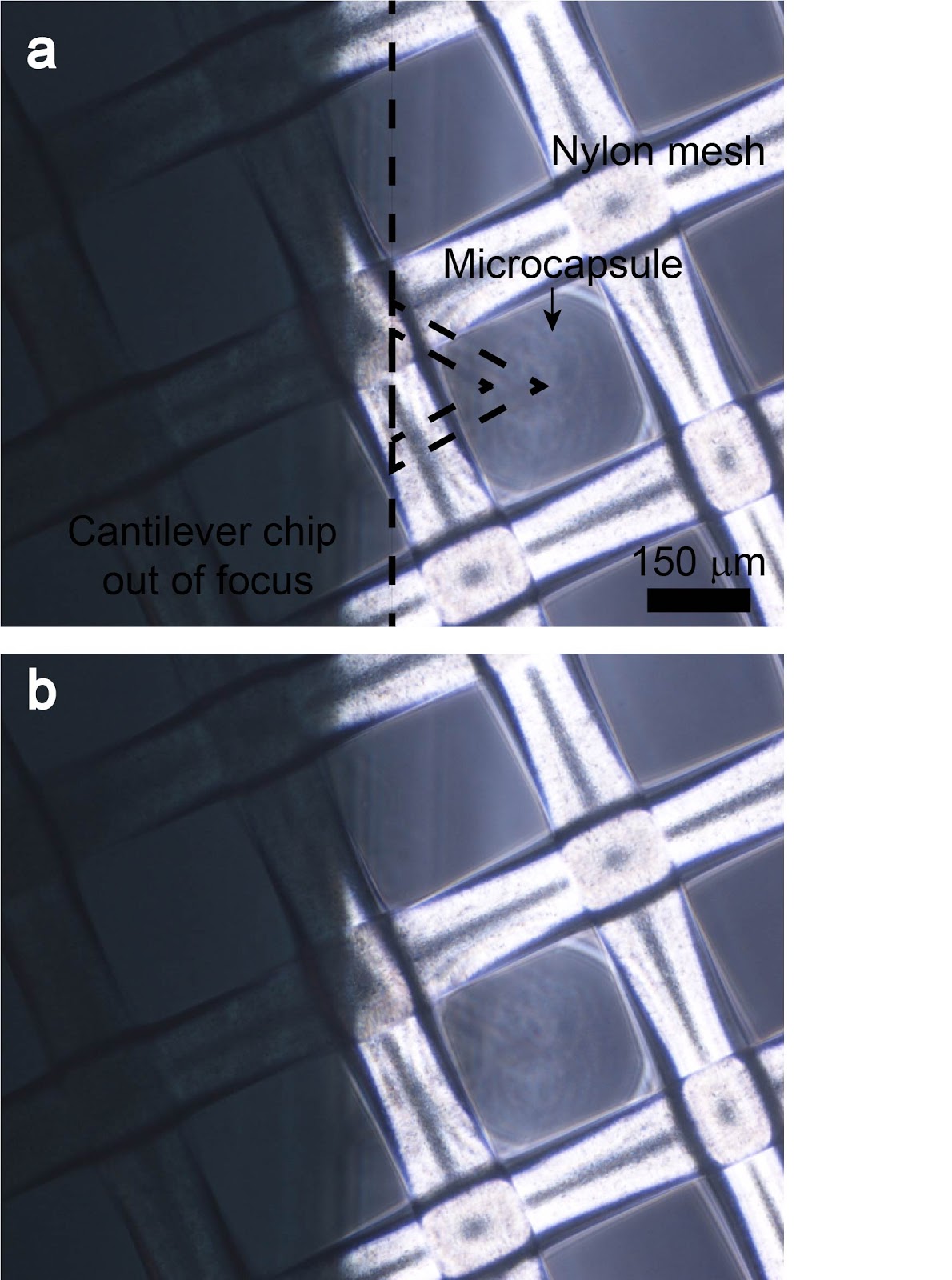
**

**Supplementary figure 1:** Brightfield image of the AFM setup. **a)** Annotated brigfield image showing one immobilized microsphere in a nylon mesh, and the cantilever aligned on the center of the image, out of focus. **b)** Non-annotated image shown in **(a).**

**
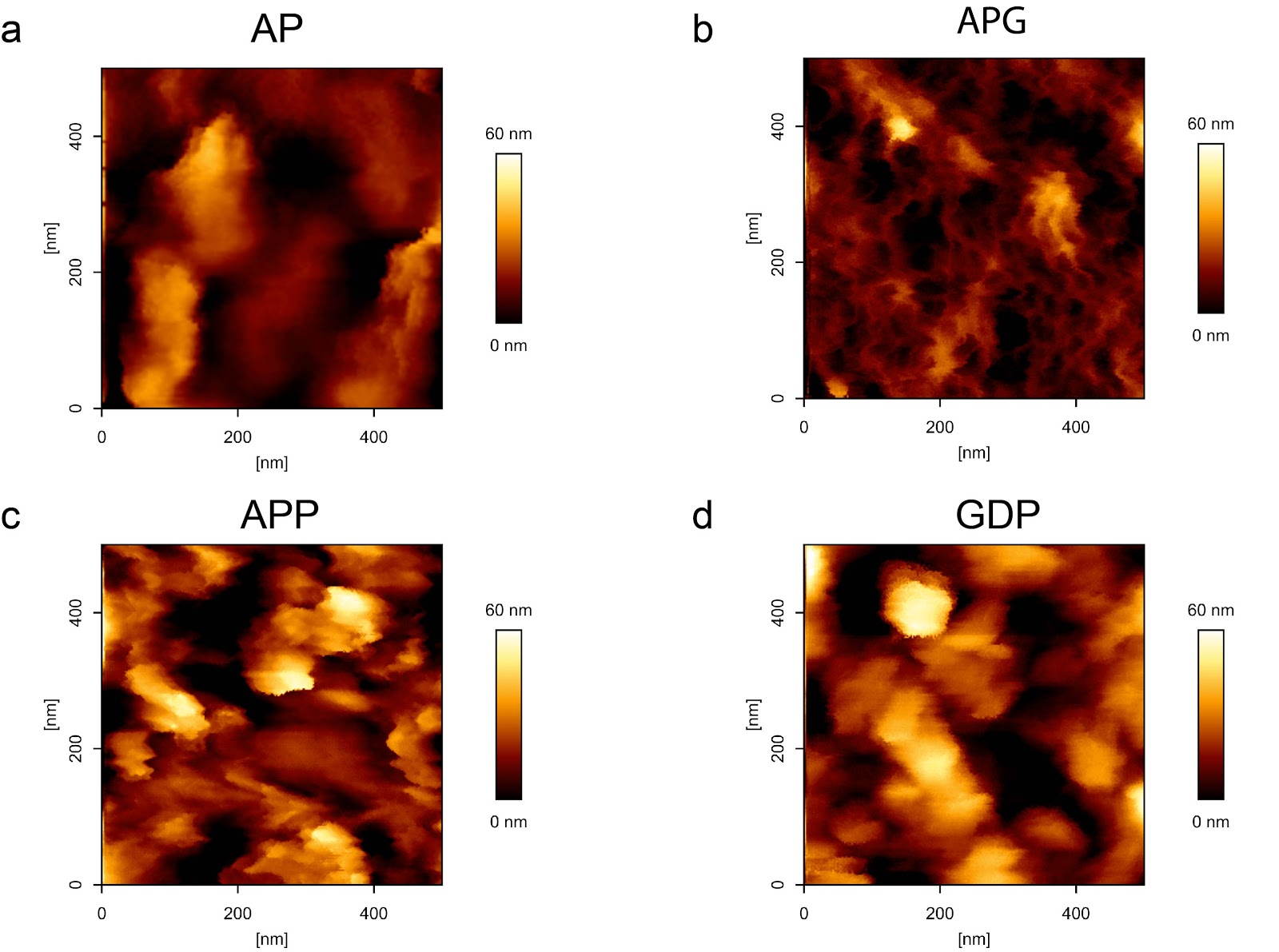
**

**Supplementary figure 2:** Representative detailed view topographies of the different microsphere compositions **a)** AP, **b)** APG, **c)** APP, **d)** GDP. The size of the topography images is 500 nm by 500 nm.

**
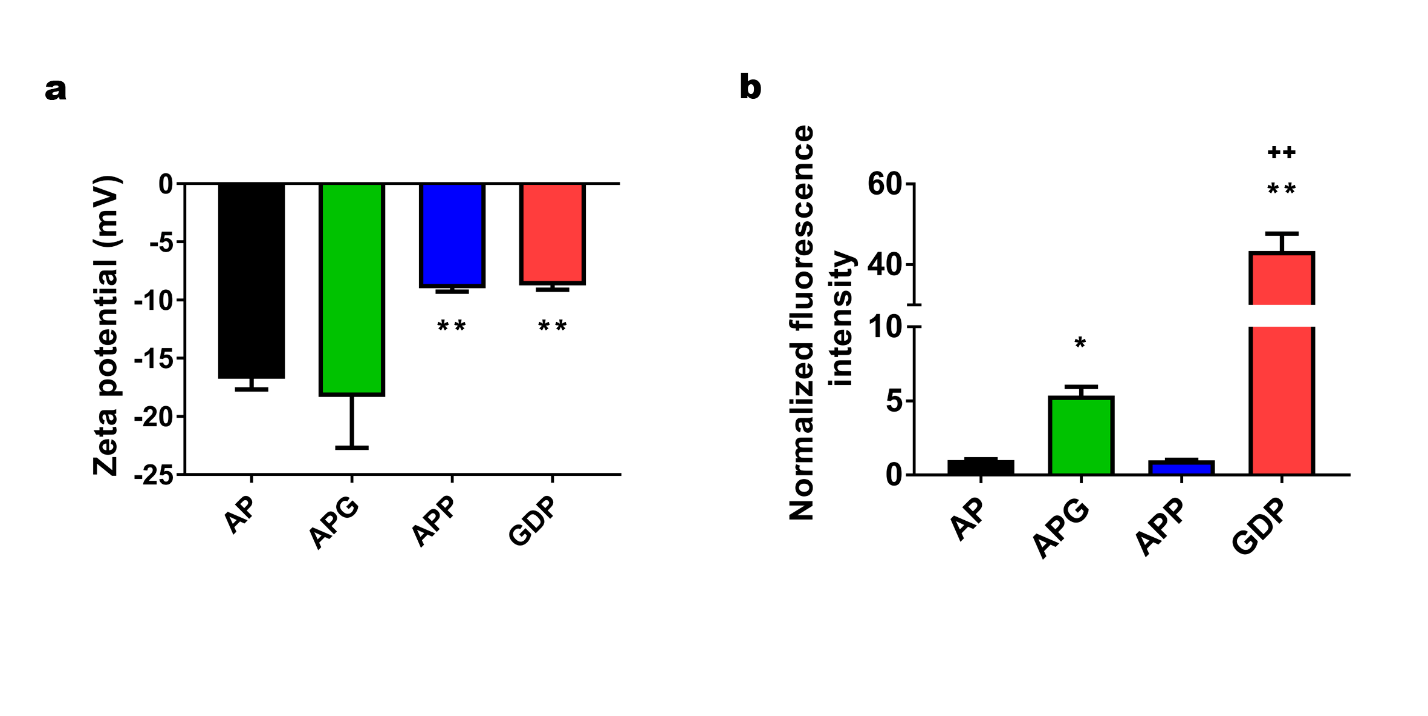
**

**Supplementary figure 3:** Validation of microsphere groups. **a)** Zeta-potential of microsphere surfaces (n = 3 samples per group). Unpaired, two-tailed t-test. **b)** Normalized fluorescence intensity of microspheres 96 h after genipin crosslinking (n = 5 samples per group). One-way ANOVA with Tamhane multiple comparison correction. Bars represent mean ± SEM. “*” indicates significance in comparison to AP. “+” indicates significance in comparison with APG.


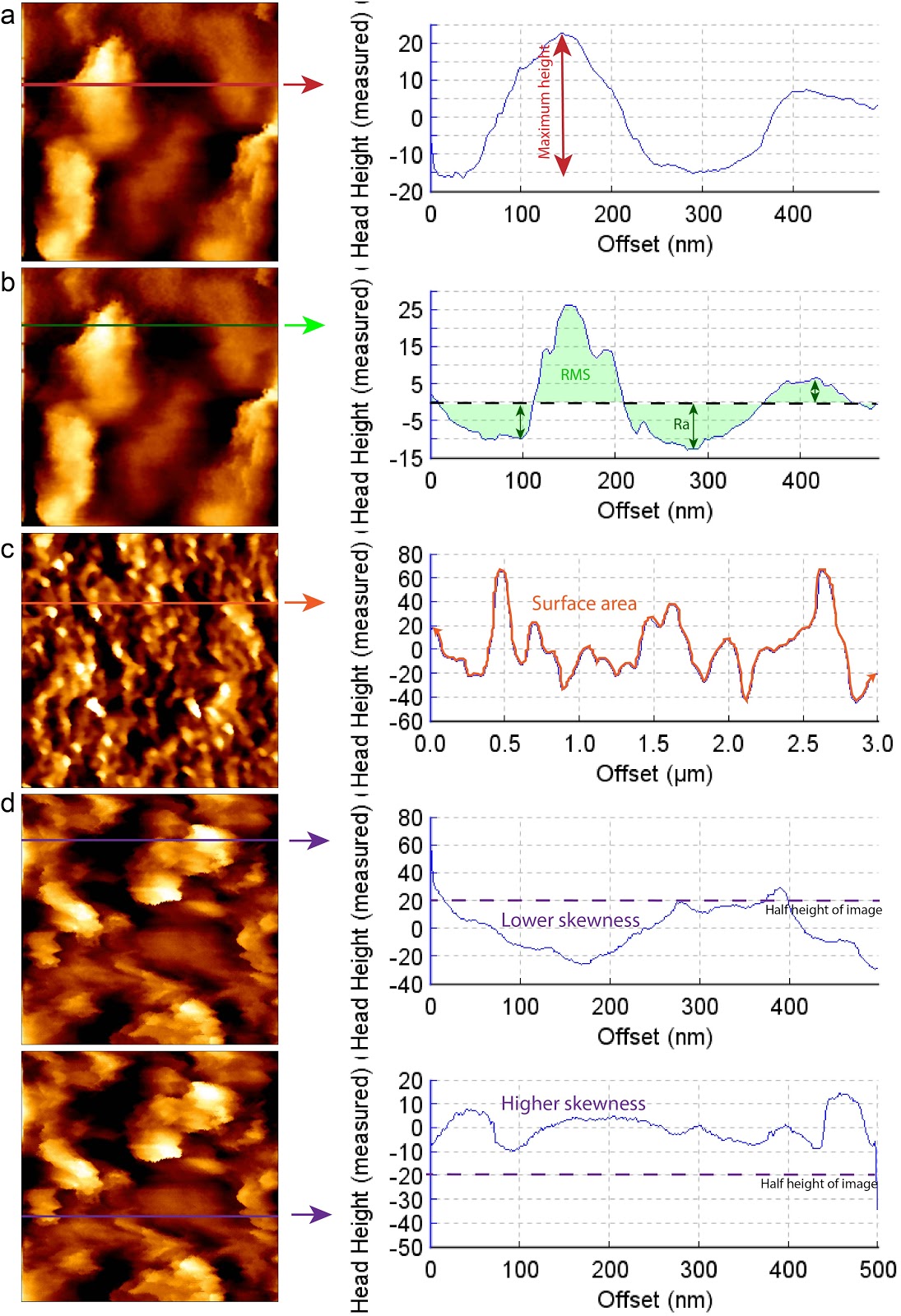


**Supplementary figure 4:** Schematic representation of the significance of topography parameters used in this article. The parameters have been represented in 2D line profiles for clarity. **a)** Representative topography of a microsphere (left) and line profile representation (right). The Maximum image height is depicted in the line profile representations. **b)** representative topography of a microsphere (left) and line profile representation indicating the physical meaning of Ra (dark green) and RMS (light green) parameters. **c)** line profile representation of surface area. **d)** From a representative topography (left) line profiles have been extracted to illustrate a lower skewness (top right) and higher skewness (bottom right).

**
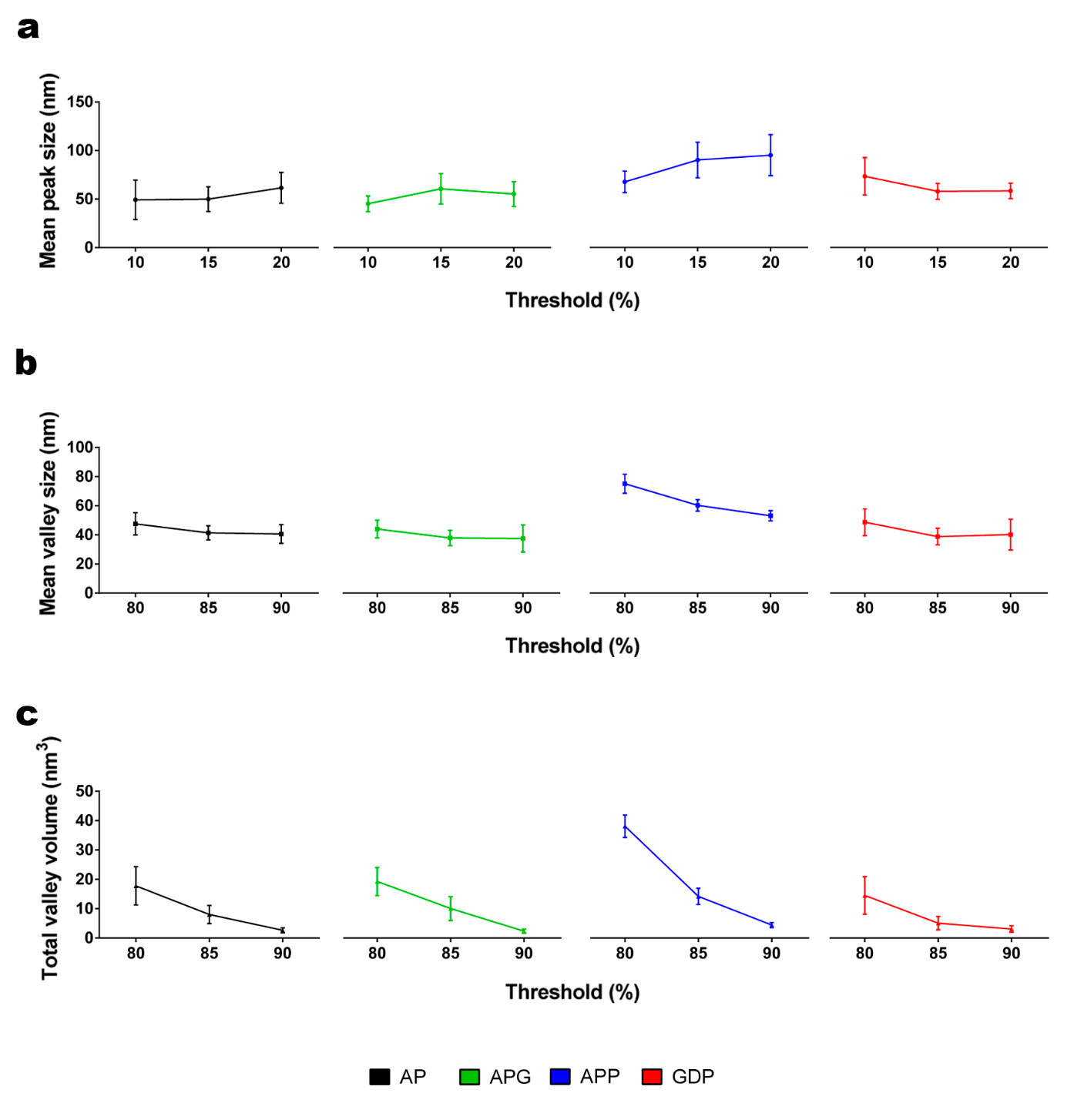
**

**Supplementary** **figure 5**:  Grain analysis performed on thresholded images. Topographies first subjected to threshold corresponding to 80 and 90% (for peak analysis) and 10**,** 20 (for valley analysis) of the maximum image height. Grain analysis was then performed to obtain **a)** mean peak diameter, **b)** mean valley diameter **c)** and total valley volume of each threshold and image type. All graphs depict mean 土 standard error of the mean.

**
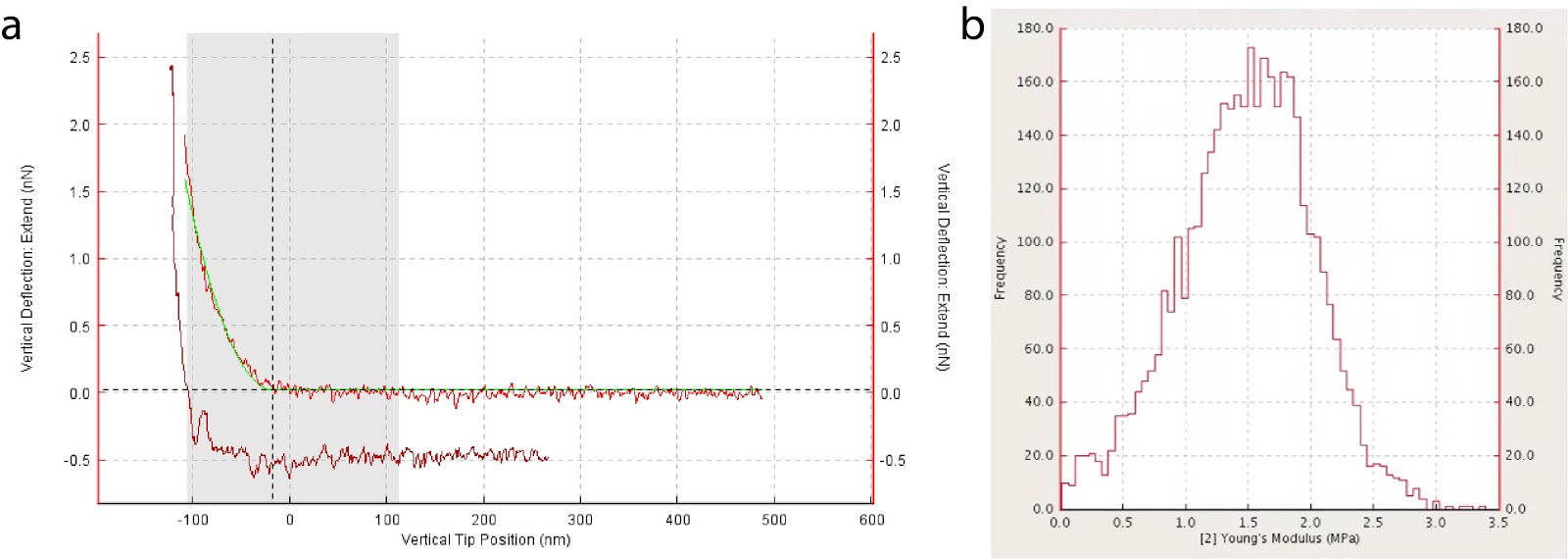
**

**Supplementary figure 6:** Representative images of the Young’s modulus analysis performed simultaneously to topography. **a)** Sample of a force spectroscopy curve corresponding to a pixel of one of the topographies acquired. Extend (bright red) and retract (dark red) curves can be observed, as well as the area used for the analysis (grey shadow) and the Hertz model fitting of the curve (green). **b)** Representative histogram of Young’s moduli acquired from one of the topographies, after analyzing each pixel as shown in (a).
